# Breast cancer dependence on MCL-1 is due to its canonical anti-apoptotic function

**DOI:** 10.1038/s41418-021-00773-4

**Published:** 2021-03-31

**Authors:** Kirsteen J. Campbell, Susan M. Mason, Matthew L. Winder, Rosalie B. E. Willemsen, Catherine Cloix, Hannah Lawson, Nicholas Rooney, Sandeep Dhayade, Andrew H. Sims, Karen Blyth, Stephen W. G. Tait

**Affiliations:** 1 CRUK Beatson Institute, Glasgow, UK; 2 Institute of Cancer Sciences, College of Medical, Veterinary and Life Sciences, University of Glasgow, Glasgow, UK; 3 CRUK Edinburgh Centre, MRC Institute of Genetics and Molecular Medicine, Edinburgh, UK; 4 Present Address: Barts Cancer Institute, Queen Mary University of London, London, UK

**Keywords:** Cancer models, Cell biology, Genetics

## Abstract

High levels of the anti-apoptotic BCL-2 family member MCL-1 are frequently found in breast cancer and, appropriately, BH3-mimetic drugs that specifically target MCL-1’s function in apoptosis are in development as anti-cancer therapy. MCL-1 also has reported non-canonical roles that may be relevant in its tumour-promoting effect. Here we investigate the role of MCL-1 in clinically relevant breast cancer models and address whether the canonical role of MCL-1 in apoptosis, which can be targeted using BH3-mimetic drugs, is the major function for MCL-1 in breast cancer. We show that MCL-1 is essential in established tumours with genetic deletion inducing tumour regression and inhibition with the MCL-1-specific BH3-mimetic drug S63845 significantly impeding tumour growth. Importantly, we found that the anti-tumour functions achieved by MCL-1 deletion or inhibition were completely dependent on pro-apoptotic BAX/BAK. Interestingly, we find that MCL-1 is also critical for stem cell activity in human breast cancer cells and high *MCL1* expression correlates with stemness markers in tumours. This strongly supports the idea that the key function of MCL-1 in breast cancer is through its anti-apoptotic function. This has important implications for the future use of MCL-1-specific BH3-mimetic drugs in breast cancer treatment.

## Introduction

The BCL-2 family regulates mitochondrial integrity to ensure appropriate activation of programmed cell death (CD) in response to developmental or physiological cues. This is achieved through balanced protein:protein interactions between pro- and anti-apoptotic family members that regulate the activation of BAX/BAK. Active BAX and BAK permeabilise the mitochondrial outer membrane leading to the release of mitochondrial intermembrane space proteins that trigger caspase activation and CD [1]. Evasion of CD is considered a hallmark of cancer, and apoptosis represents a major barrier to cellular transformation and oncogenic progression [2]. Resistance to apoptosis in cancer can be achieved through disruption in the balance of BCL-2 proteins to enhance pro-survival functions. The paradigm for this is aberrant BCL-2 expression through chromosomal translocation in follicular lymphoma though increased anti-apoptotic BCL-2 protein expression occurs across diverse cancer types through mechanisms including gene amplification, increased transcription or translation and protein stability [3, 4]. Apoptotic sensitivity can be restored by chemical mimicry of the pro-apoptotic BCL-2 proteins with so-called BH3-mimetic drugs. Neutralisation of elevated pro-survival BCL-2 family members has demonstrable clinical impact in cancer; venetoclax (ABT199) is a BCL-2-specific BH3-mimetic that has single-agent and combinatorial efficacy in some types of lymphoma and BH3-mimetic drugs are now in clinical trials for a range of cancers [5–8].

BH3-mimetic drugs that specifically target MCL-1 have been developed and entered clinical trials for the treatment of haematopoietic malignancies. In addition to its prominent role in haematopoietic cancers, MCL-1 has emerged as a key player in breast cancer with high levels of MCL-1 protein in primary breast cancer samples correlating with poor patient prognosis [9–16]. There are compelling data showing that targeting MCL-1 represents a therapeutic opportunity in breast cancer with model systems revealing that breast cancer may be dependent on MCL-1, and that MCL-1 inhibition can enhance the effect of conventional cancer therapies [9–12, 16–19]. Importantly, numerous non-apoptotic roles for MCL-1 have also been described that may also be relevant to cancer. These include regulation of mitochondrial dynamics, oxidative phosphorylation, reactive oxygen species, autophagy, pluripotency, long chain fatty acid synthesis and the DNA damage response [20–28]. Importantly, not all of these functions can be blocked by BH3-mimetic drugs implying that additional approaches to target MCL-1 may be required for maximal therapeutic impact (reviewed in [29]). Addressing this key point, we set out to determine whether fully established tumours in an immune competent breast cancer model were dependent on MCL-1 and, if so, whether this was due to anti-apoptotic MCL-1 activity and/or its non-apoptotic functions.

We find that acute genetic deletion or pharmaceutical targeting of MCL-1 significantly impedes the growth of established MMTV-PyMT mammary tumours in vivo. Crucially, this oncogenic function of MCL-1 was completely dependent upon its anti-apoptotic function because loss of pro-apoptotic BAX and BAK completely prevented the effect of MCL-1 loss. Importantly, MCL-1 was required for breast cancer stem cell function in vitro and this was also due to MCL-1’s canonical anti-apoptotic function since it could be ablated by deletion of BAX/BAK and targeted with MCL-1-specific BH3-mimetic drugs. These data strongly support the premise of MCL-1 inhibition as an adjunct to breast cancer therapy and highlight that the major role of MCL-1 in breast cancer is via inhibition of apoptosis.

## Results

### MCL-1 is required for tumour maintenance in vivo

**Fig. 1:**
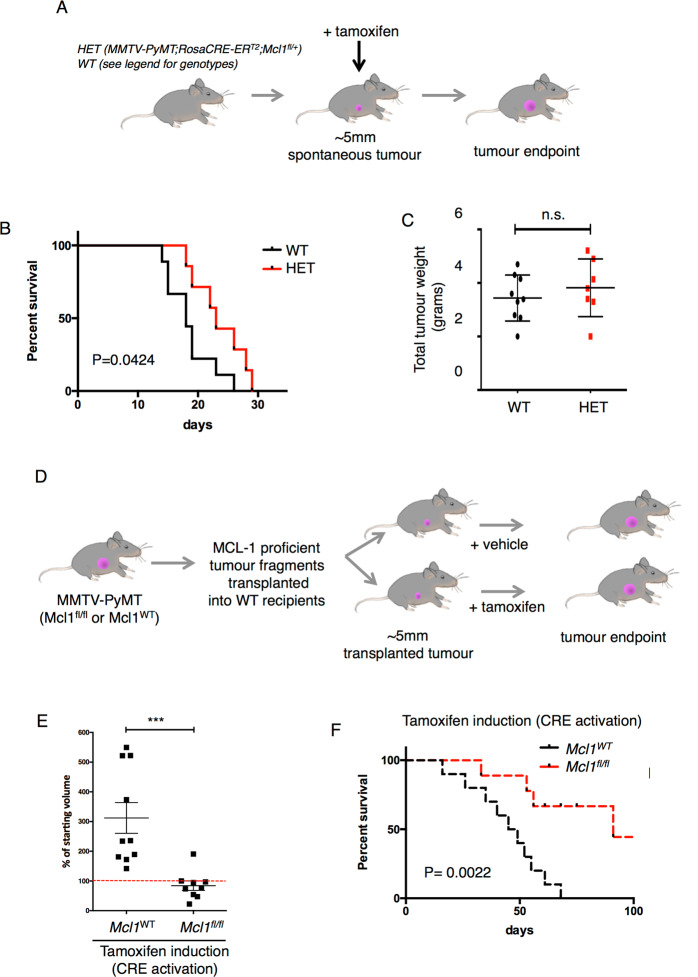
*MCL-1* is required for *MMTV-PyMT* tumour maintenance in vivo. **A** Diagram of experimental set-up for results presented in **B**, **C**. Female *MMTV-PyMT* mice were monitored until the largest tumour reached 5 mm diameter, induced with tamoxifen (day 0 on graph) for 4 consecutive days (to induce recombination in mice carrying *RosaCRE-ER*^*T2*^*;Mcl1*^*fl/+*^ alleles) and monitored until clinical endpoint. **B** Kaplan–Meier survival graph of mice WT for *Mcl1* (*n* = 9) (median survival 18 days) and HET (*n* = 7) (median survival 23 days) post tamoxifen induction (*P* = 0.0424). Log-rank (Mantel–Cox) test. Genotypes of WT mice were 2× *MMTV-PyMT;RosaCRE-ER*^*T2*^*;Mcl1*^*+/+*^;*Rosa*^*LSL-RFP*^, 4× *MMTV-PyMT;Mcl1*^*fl/+*^;*Rosa*^*LSL-RFP*^ and 3× *MMTV-PyMT;Mcl1*^*fl/fl*^;*Rosa*^*LSL-RFP*^, genotypes of seven HET mice were *MMTV-PyMT;RosaCRE-ER*^*T2*^*;Mcl1*^*fl/+*^;*Rosa*^*LSL-RFP*^. **C** Total tumour weight at endpoint of mice shown in **B**. WT (*n* = 9), HET (*n* = 7), n.s. not significant, unpaired *t* test. **D** Diagram of experimental set-up for results presented in **E**, **F** (see also Supplementary Fig. 1). Tumours were harvested from three ‘*Mcl1*^*WT*^’ (genotypes were *MMTV-PyMT;RosaCRE-ER*^*T2*^*;Mcl1*^*+/+*^;*Rosa*^*LSL-RFP*^*/MMTV-PyMT;Mcl1*^*fl/+*^;*Rosa*^*LSL-RFP*^*/MMTV-PyMT;Mcl1*^*fl/fl*^;*Rosa*^*LSL-RFP*^) and 3 ‘*Mcl1*^*fl/fl*^’ (genotypes were 2× *MMTV-PyMT;RosaCRE-ER*^*T2*^*;Mcl1*^*fl/fl*^;*Rosa*^*LSL-RFP*^ and 1× *MMTV-PyMT;RosaCRE-ER*^*T2*^*;Mcl1*^*fl/fl*^) donor mice and tumour fragments orthotopically transplanted into multiple FVB recipients (ten recipients of *Mcl1*^WT^ fragments and nine recipients of *Mcl1*^*fl/fl*^ fragments). Transplanted tumours were monitored until ~5 mm diameter before tamoxifen-induced CRE-ER^T2^ activation. **E** Change in tumour volume 3 weeks post *Mcl1* deletion. Error bars represent mean ± SD, ***P = 0.0009 unpaired *t* test. Red dotted line indicates 100% of starting volume (i.e., no change). No *Mcl1*^WT^ tumours regressed and 8/9 *Mcl1*^*fl/fl*^ tumours regressed following CRE activation. **F** Kaplan–Meier survival curve of mice carrying tumours shown in (**E**). Median survival *Mcl1*^WT^ 47 days*, Mcl1*^*fl/fl*^ 91 days post tamoxifen induction *P* = 0.0022 Log-rank (Mantel–Cox) test.

Having previously demonstrated an essential genetic requirement for MCL-1 in tumour development in the *MMTV-PyMT* mammary model we wanted to mimic therapeutic targeting of MCL-1 in an adjuvant setting by determining the impact of *Mcl1* deletion in established tumours [16]. *MMTV-PyMT* mice were crossed with *RosaCRE-ER*^*T2*^;*Mcl1*^*fl/+*^ mice and cohorts aged until mammary tumours reached 5 mm diameter at which time ubiquitous deletion of one allele of *Mcl1* was achieved by tamoxifen administration and tumour growth monitored until clinical endpoint (Fig. 1A). Interestingly, inactivation of just one allele of *Mcl1* in these mice (denoted HET), at a time when palpable tumours had already established, was sufficient to restrict tumour growth and significantly extend survival (Fig. 1B). Importantly, the total tumour burden for mice wild type (WT) and HET for *Mcl1* was comparable at endpoint despite HET mice surviving significantly longer (Fig. 1C and Supplementary Fig. 1A). Having shown that acute deletion of just one allele of *Mcl1* impedes the growth of established tumours in vivo we investigated the impact of targeting both alleles of *Mcl1*. To circumvent lethality due to the requirement for MCL-1 in haematopoietic stem cells and cardiomyocytes, we used a tumour fragment transplantation system where *MMTV-PyMT* tumours harbouring either WT or floxed alleles of *Mcl1* are transplanted into syngeneic recipients prior to tamoxifen-activated *CRE-ER*^*T2*^ recombination (Fig. 1D) [22, 23, 30]. In this way, fragments of WT and *Mcl1*^*fl/fl*^ (MCL-1 proficient) tumours were engrafted into the mammary fat pad of multiple WT recipients and allowed to establish before tamoxifen induction to delete both alleles of *Mcl1* in *Mcl1*^*fl/fl*^ tumours. Remarkably, homozygous *Mcl1* deletion induced tumour regression in 8/9 *Mcl1*^*fl/fl*^ tumours but no WT tumour regressed (Fig. 1E). This tumour-repressive effect of *Mcl1* deletion was sustained and median survival increased from 47 to 91 days (*P* = 0.0022) relative to WT controls with 4/9 recipients of *Mcl1*^fl/fl^ tumours surviving long term with no palpable tumour remaining (Fig. 1F). In contrast, tumour growth and survival of WT and *Mcl1*^*fl/fl*^ tumours was similar in the absence of active CRE (Supplementary Fig. 1B, C). Together these results reveal that MCL-1 is an essential gene in established tumours and that targeting MCL-1 at clinically actionable stages of tumour development can result in tumour regression with long-term impact on survival.

### MCL-1 inhibition significantly impedes growth of *MMTV-PyMT* tumours in vivo

**Fig. 2:**
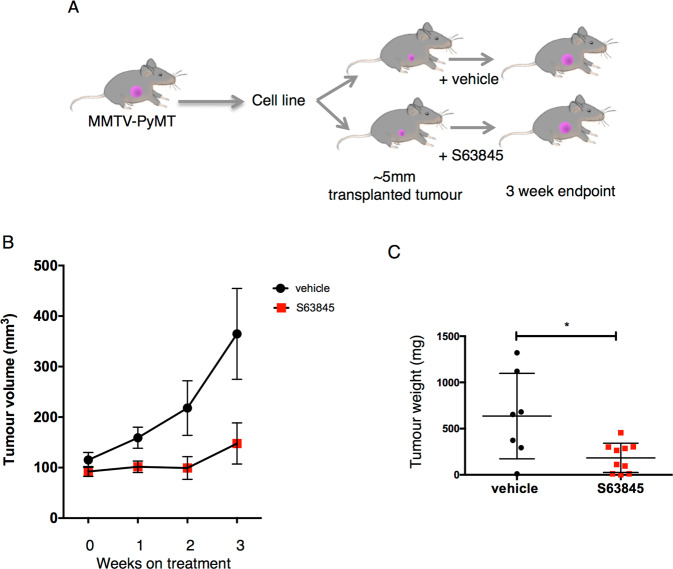
MCL-1 inhibition restricts growth of established *MMTV-PyMT* tumours in vivo. **A** Diagram of experimental set-up for results presented in **B**, **C**. *MMTV-PyMT* cell line (CRISPR/Cas9 control, described in Supplementary Fig. 2) was injected into mammary fat pad of FVB recipients and tumours were allowed to grow to ~5 mm before assignment to vehicle or S63845 treatment groups. S63845 treatment was at 25 mg/kg IV twice per week for 3 weeks and tumour volume monitored. **B** Tumour growth is suppressed upon treatment with S63845, individual tumours were measured thrice weekly and points represent weekly mean ± SE. Vehicle control (*n* = 7) mice and S63845 (*n* = 10) mice. **C** Tumour weight at end of experiment following 3 weeks of vehicle (*n* = 7) or S63845 treatment (*n* = 10). Graph shows mean ± SD, unpaired *t* test. *P = 0.0112.

Our genetic models of acute *Mcl1* deletion provide strong rationale for MCL-1 as a therapeutic target in established breast cancers. Recent pharmaceutical advances have led to the development of BH3-mimetic MCL-1 inhibitors suitable for in vivo use [17, 31–33]. We therefore wished to further model targeting MCL-1 in a breast cancer therapy setting and test whether inhibition of MCL-1 with a BH3-mimetic drug would impact on clinically palpable mammary tumours. To this end, we used a syngeneic orthotopic transplantation model (Fig. 2A, *MMTV-PyMT;CRISPR/Cas9* control cells described in Supplementary Fig. 2A). When tumours reached 5 mm diameter, recipient mice were randomly assigned to vehicle or MCL-1 inhibitor (S63845) groups and underwent twice weekly treatment for 3 weeks. MCL-1 inhibition with S63845 led to a significant impairment of tumour growth (Fig. 2B and Supplementary Fig. 2B) with reduction in post-treatment tumour weight to around one third when compared to vehicle treated controls (mean of 636 mg down to 184 mg, *P* = 0.0112, Fig. 2C). Interestingly, once weekly treatment with 25 mg/kg S63845 with or without the conventional chemotherapeutic doxetaxel did not restrict expansion of *MMTV-PyMT* tumour cells in vivo. This suggests that prolonged inhibition of MCL-1 is required for a therapeutic effect and cannot be significantly substituted by conventional chemotherapy in this setting (Supplementary Fig. 2C, D). Thus, at appropriate dosing regimens, pharmaceutical targeting of MCL-1 with BH3-mimetics such as S63845 can act as a single-agent therapy to slow tumour growth in a syngeneic mouse model of breast cancer.

### Breast cancer dependency on MCL-1 is lost in the absence of BAX and BAK

**Fig. 3:**
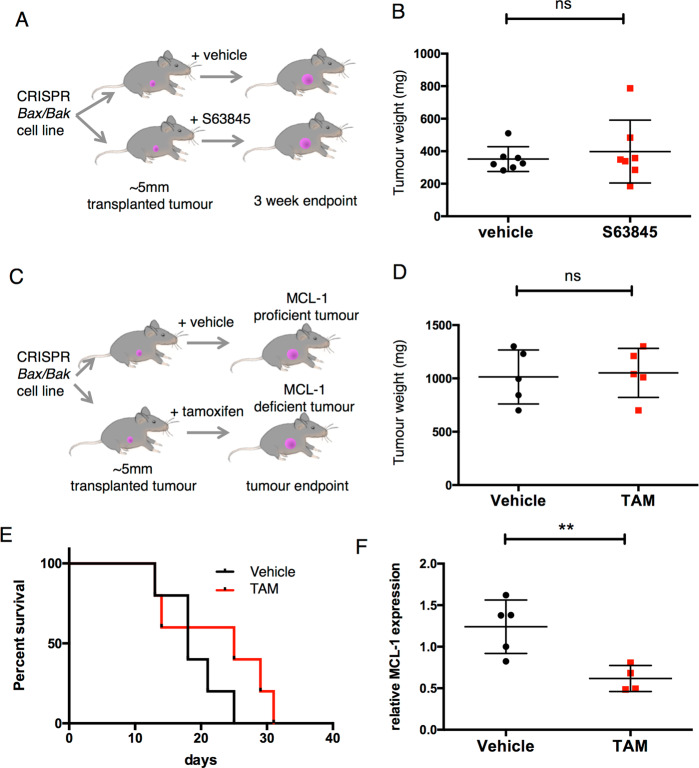
MCL-1 dependence requires BAX/BAK. **A** Diagram of experimental set-up for results presented in **B**. *MMTV-PyMT* CRISPR/Cas9 *Bax/Bak* cell line (derivation described in Supplementary Fig. 2A) was injected into mammary fat pad of FVB recipients and tumours allowed to grow to ~5 mm before assignment to vehicle or S63845 treatment groups. S63845 treatment was at 25 mg/kg IV twice per week for 3 weeks. **B** Mean tumour weight of mice described in **A**, following 3 weeks of vehicle control (*n* = 7) or S63845 (*n* = 7) treatment (25 mg/kg IV twice per week) is shown (±SD), n.s. not significant unpaired *t* test. **C** Diagram of experimental set-up for results presented in **D**–**F**. *MMTV-PyMT; CRISPR/Cas9 Bax/Bak* tumour cell line (as used in **A**, **B**, this cell line carries *RosaCRE-ER*^*T2*^*;Mcl1*^*fl/fl*^ alleles that were not activated in **A**, **B**, derivation described in Supplementary Fig. 2A) was injected into mammary fat pad of FVB recipients and tumours allowed to grow to ~5 mm before assignment to vehicle or tamoxifen groups (tamoxifen induces CRE-ER^T2^-mediated deletion of *Mcl1*^*fl/fl*^); therefore, vehicle treated tumours will be MCL-1 proficient and tamoxifen-induced tumours (TAM) will be MCL-1 deficient. Tumour size was monitored by calliper measurement thrice weekly until harvesting at predefined ethical endpoint (15 mm diameter). **D** Points indicate individual tumour weights at endpoint, vehicle control (*n* = 5) or tamoxifen (*n* = 5). Error bars represent mean ± SD, n.s. not significant unpaired *t* test. **E** Kaplan–Meier graph showing tumour-related survival of mice shown in **D**. Day 0 indicates vehicle/tamoxifen induction (at tumour diameter ~5 mm). *Mcl1* deletion in established tumours does not alter survival when *Bax/Bak* have been CRISPR/Cas9 targeted. Vehicle control (*n* = 5) or tamoxifen (*n* = 5). *P* = 0.2482 Log-rank (Mantel–Cox) test. **F** Western blot quantification shows sustained decrease in MCL-1 from endpoint tumours shown in **D**, **E**. Tumour material sampled for western blot will include host-derived (WT for *Mcl1*) stroma and donor-derived tumour epithelium. MCL-1 expression is calculated relative to β-actin control for each sample, each point represents an individual tumour and error bars represent mean ± SD. **P < 0.01, unpaired *t* test. Western blots shown in Supplementary Fig. 3B.

Non-apoptotic functions for MCL-1 have been described in cardiomyocytes, pluripotent stem cells, breast cancer stem cells and neurons [13, 21–23, 26–28]. To determine whether the conventional anti-apoptotic role of MCL-1 was responsible for mammary tumour sensitivity to MCL-1 loss/inhibition we deleted *Bax* and *Bak* to bypass the canonical anti-apoptotic function of MCL-1 (regulation of mitochondrial outer membrane permeabilization). Following orthologous transplant of *Bax/Bak* CRISPR/Cas9 edited *MMTV-PyMT* cells into WT recipients, tumours were allowed to grow to 5 mm diameter before treatment with MCL-1 inhibitor S63845 or vehicle control (Fig. 3A and Supplementary Figs. 2A and 3A). Strikingly, in tumours that had been engineered for BAX/BAK deficiency, S63845 treatment had no impact on tumour growth (Fig. 3B). This is in contrast to the pronounced impact of S63845 we observed in the paired BAX/BAK-proficient *MMTV-PyMT* cells (Fig. 2B, C and Supplementary Fig. 2A). Whilst we had shown that *Mcl1* deletion can result in long-term tumour suppression (Fig. 1F), genetic deletion of *Mcl1* had no effect on tumour growth in BAX/BAK-deficient tumours (Fig. 3C–E) and unlike BAX/BAK-proficient *MMTV-PyMT* tumours, decreased MCL-1 expression was still apparent in the context of BAX/BAK loss (Fig. 3F and Supplementary Fig. 3B–D) [16].

### MCL-1 is essential for *MMTV-PyMT* cancer stem cell viability

**Fig. 4:**
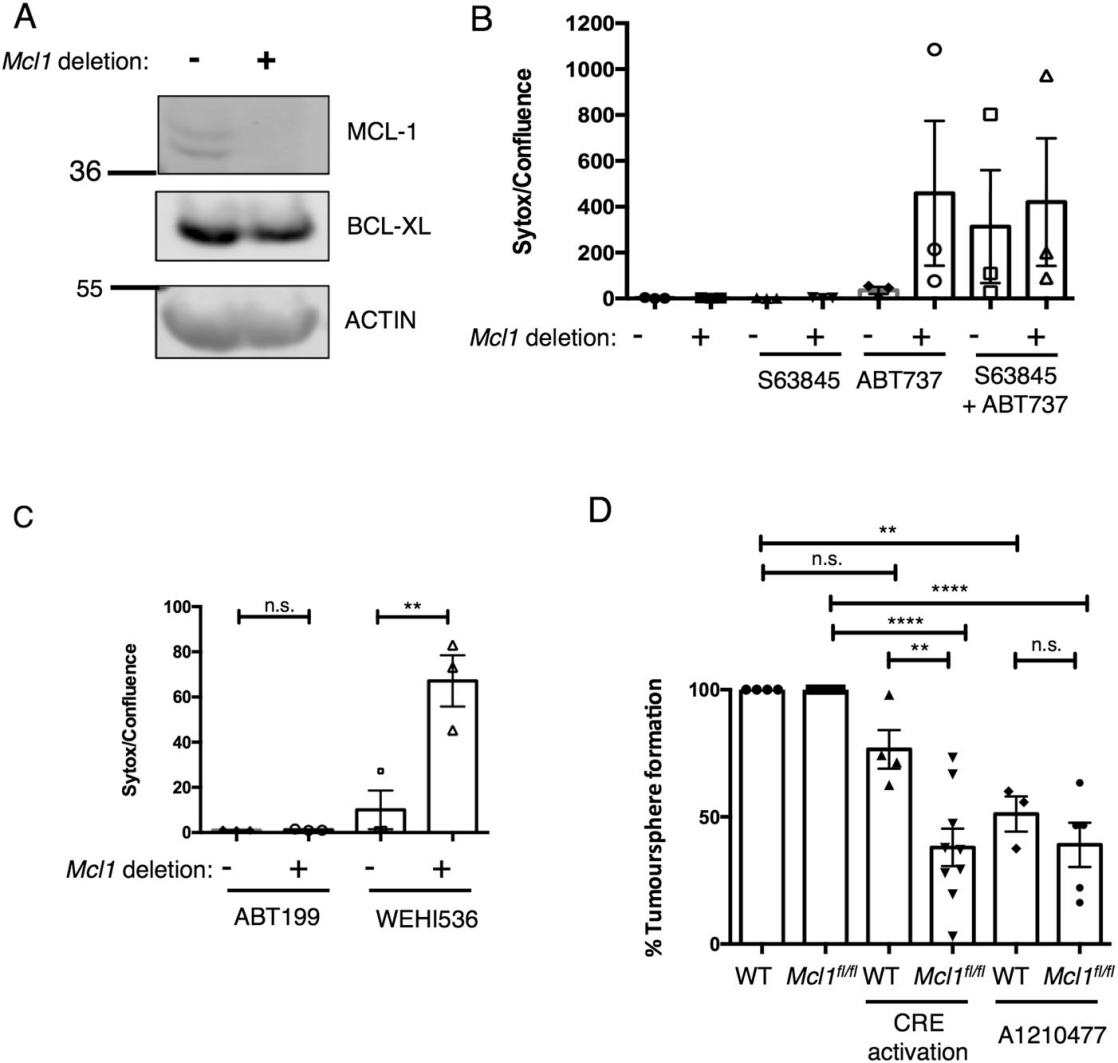
MCL-1 is essential for *MMTV-PyMT* breast cancer stem cells. **A** Western blot showing impact of CRE activation on MCL-1 and BCL-XL expression in a cell line from *MMTV-PyMT;RosaCRE-ER*^*T2*^*;Mcl1*^*fl/fl*^ tumour. CRE activated in vitro with 100 nM 4-hydroxytamoxifen (4-OHT), samples harvested after 72 h. MCL-1 is efficiently deleted, BCL-XL level is not increased. **B** Genetic deletion or pharmacological inhibition of MCL-1 does not inhibit tumour cell line viability in 2D culture. *Mcl1* deletion induced with 100 nM 4-OHT at time of plating, medium changed 24 h later and 1 μM of indicted BH3-mimetic drugs added for an additional 24 h before quantification of cell death as expressed by Sytox/Confluence. Cells plated in triplicate wells and repeated three times. Graph shows mean ± SD, no significant differences as determined by one-way ANOVA with Tukey’s correction for multiple comparisons. Cell line derived from *MMTV-PyMT;RosaCRE-ER*^*T2*^*;Mcl1*^*fl/fl*^ tumour. **C** *MMTV-PyMT* cell lines show co-dependence on MCL-1 and BCL-XL in 2D culture. Induction of *Mcl1* deletion with 100 nM 4-OHT at time of plating, medium changed 24 h later and 1 μM of indicted BH3-mimetic drugs added for a further 24 h prior to determining viability by Sytox/Confluence. Cells plated in triplicate, for three experiments, data points represent mean of individual experiments (two independent cell lines from *MMTV-PyMT;RosaCRE-ER*^*T2*^*;Mcl1*^*fl/fl*^ tumours contributed to these data points (lines 3.1j and 5.1e with 5.1e being tested twice). Graph shows mean ± SD, **P < 0.01 determined by one-way ANOVA with Tukey’s correction for multiple comparisons. **D** MCL-1 deletion or inhibition impairs cancer stem cell activity. *MMTV-PyMT* cell lines were plated as single-cell suspensions in a tumoursphere assay and inducer/inhibitor (4-OHT or A1210477) added at time of plating. *CRE-ER*^*T2*^ was activated in vitro with 100 nM 4-OHT (‘CRE activation’) to delete *Mcl1* in *Mcl1*^*fl/fl*^, cells or MCL-1 inhibited with 1 µM A1210477 at time of plating. Tumoursphere count on day 7 is expressed as percentage relative to untreated control. Points represent mean tumoursphere count relative to control condition for each cell line. WT tumour cell line (3.3 h) is *MMTV-PyMT;RosaCRE-ER*^*T2*^*;Mcl1*^*+/+*^, experiment was set up a total of four independent times with 4-OHT and A1210477 treatment carried out on three of those repeats. *Mcl1*^*fl/fl*^ data points come from two independent *MMTV-PyMT;RosaCRE-ER*^*T2*^*;Mcl1*^*fl/fl*^ tumour cell lines, line 3.1j was set up with 4-OHT five independent times and A1210477 treatment carried out on three of those repeats and line 5.1e was set up four independent times with 4-OHT and A1210477 treatment carried out two repeats. Graph shows mean ± SE. **P < 0.01, ****P < 0.0001, one-way ANOVA analysis with Tukey’s correction for multiple comparisons.

Previous studies have grouped human breast cancer cell lines into MCL-1-dependent and MCL-1-independent groups based on in vitro sensitivity to MCL-1 inhibition or knockdown in 2D monolayer assays [9, 12, 34]. To our surprise, the dramatic impact of loss or inhibition of MCL-1 in *MMTV-PyMT* tumours that we observed in vivo was not recapitulated in vitro with acute deletion of MCL-1 in cell lines derived from *MMTV-PyMT* primary tumours (hereafter referred to as tumour cell lines) (Fig. 4A, B and Supplementary Fig. 4A). Moreover, treatment with the MCL-1-specific BH3-mimetic drug S63845 alone failed to overtly affect viability when tumour cell lines were maintained in 2D monolayers (Fig. 4B and Supplementary Fig. 4A). In 2D culture these tumour cell lines are however still dependent on pro-survival BCL-2 proteins as combined targeting of MCL-1 (genetically or pharmaceutically with S63845) and BCL-2/BCL-XL (with ABT737) resulted in high, but variable, levels of CD (Fig. 4B, also observed in an additional independently derived tumour cell line in Supplementary Fig. 4A). Parsing of the relative contribution of BCL-2 and BCL-XL with additional BH3-mimetic drugs ABT199 (BCL-2 specific) and WEHI536 (BCL-XL specific) revealed that together with MCL-1, BCL-XL (rather than BCL-2) is required for tumour cell line monolayer viability and this is not due to compensatory upregulation of BCL-XL in the absence of MCL-1 (Fig. 4A, C).

In contrast to the indifference of these tumour cell lines to MCL-1 deletion or inhibition in 2D adherent culture, there was a strong effect in 3D-tumoursphere culture. An important role for MCL-1 in breast cancer stem cells has been suggested and we reasoned that the bulk of cells growing in vitro in monolayers might not represent the cells driving tumour maintenance in vivo such as cancer stem cells [13]. To address this, a tumoursphere assay in non-adherent culture was utilised that is known to reflect breast cancer stem cell activity [35]. This revealed a strong genetic requirement for MCL-1 in breast cancer stem cells (Fig. 4D), which was pharmacologically confirmed using the MCL-1-specific BH3-mimetic drug A1210477 (Fig. 4D). Therefore, whilst MCL-1 may be dispensable for bulk *MMTV-PyMT* cell culture in 2D monolayer, it is essential for growth of breast cancer stem cells in vitro.

### MCL-1 is required for human breast cancer stemness via BAX/BAK

**Fig. 5:**
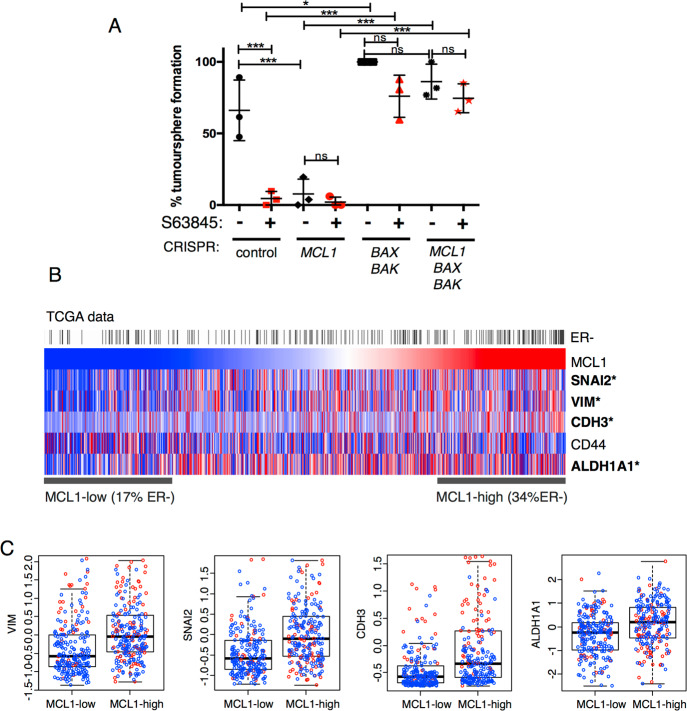
MCL-1 dependence requires BAX/BAK. **A** Tumoursphere assay with indicated CRISPR/Cas9-modified MDA-MB-231 cell lines, 1 µM S63845 was added at time of plating and spheres were counted at day 7. Points represent independent experiments expressed as % tumoursphere formation relative to untreated BAX/BAK-deficient control. Bars represent mean ± SD of three biological replicates. One-way ANOVA analysis with Tukey’s correction for multiple comparisons. *P < 0.05, ***P < 0.001, n.s. not significant. **B** Correlation of *MCL1* expression with*, SNAI2, VIM, CDH3, CD44* and *ALDH1A1* across TCGA breast cancers (979 tumours [52]). *Pearson correlation *P* < 0.05. ER-negative status indicated by black bar in top row. **C** Comparison of *VIM, SNAI2, CDH3* and *ALDH1A1* expression in low *MCL1* (lower quartile) and high *MCL1* (upper quartile) with ER status indicated (red dots for ER-negative, blue dots for ER-positive tumours) TCGA dataset as in **B**.

Given the profound requirement for MCL-1 in tumours and cancer stem cells in the mouse *MMTV-PyMT* model, we wished to interrogate the relevance of these findings to human breast cancer. The MDA-MB-231 human triple-negative breast cancer cell line shows properties of breast cancer stem cells but in 2D monolayer is insensitive to loss or inhibition of MCL-1 [9, 12, 34]. Intriguingly, we found that in tumoursphere assay conditions MDA-MB-231 cells were highly sensitive to MCL-1 inhibition with BH3-mimetic drugs A1210477 and S63845 (Supplementary Fig. 5A). To further test the requirement for canonical MCL-1 function in human breast cancer stem cells we therefore used CRISPR/Cas9 editing to generate *MCL1*-, *BAX/BAK*- and *MCL1/BAX/BAK*-deficient versions of the human breast adenocarcinoma MDA-MB-231 cell line (Supplementary Fig. 5B). As expected, genetic targeting of MCL-1 was tolerated without impacting on viability when these cell lines were maintained in 2D culture ([9, 12, 34] and data not shown). MDA-MB-231 control cells were highly sensitive to MCL-1 inhibition with S63845 in the tumoursphere assay and *MCL1*-deficient cells were unable to form tumourspheres in vitro confirming a specific role for MCL-1 in breast cancer stem cell function (Fig. 5A). Importantly, this was completely restored upon co-deletion of *BAX/BAK* (Fig. 5A). The ability of MCL-1 inhibitor S63845 to restrict human breast cancer stem cell growth was also negated in the absence of BAX/BAK, clearly illustrating the importance of canonical anti-apoptotic MCL-1 function in breast cancer stem cells (Fig. 5A). Together with our experiments utilising mouse models, these data reveal that MCL-1 function in breast cancer stem cells in vitro and in tumour survival in vivo is due to its anti-apoptotic function that is targetable by BH3-mimetic drugs.

In order to probe whether MCL-1 might be specifically associated with breast cancer stemness across human breast cancers we examined a number of tumour gene expression datasets. Importantly, MCL-1 expression was found to be significantly correlated with stemness markers *SNAI2*, *VIM*, *CDH3*, *CD44* and *ALDH1A1* (Fig. 5B and Supplementary Fig. 5C, E, F). Splitting tumours into *MCL1*-low and *MCL1*-high groups confirmed that these stemness markers have elevated expression in *MCL1*-high tumours, which were also significantly enriched for ER-negative tumours (Fig. 5C and Supplementary Fig. 5D). Similar results were also found for three other datasets, representing a combined total of over 9000 breast tumours (Supplementary Fig. 5C–F). Together, these data suggest that MCL-1 may be an important player in maintaining stem cell populations of primary breast cancers.

## Discussion

In this study we provide evidence that targeting MCL-1 could have therapeutic impact in established breast cancers and show that the mechanism of breast cancer dependence on MCL-1 is through its canonical anti-apoptotic function that can be targeted by MCL-1-specific BH3-mimetics.

MCL-1 inhibitors are in clinical development with a focus on haematopoietic cancers including multiple myeloma and acute myeloid leukemia [36]. Using genetic methods, we have previously shown that MCL-1 is required for tumour development in a mouse model of breast cancer and a number of groups have shown the importance of MCL-1 for breast cancer cell line survival in vitro through genetic and pharmaceutical targeting [9–12]. Despite these encouraging findings, pharmaceutical targeting of MCL-1 as a single agent has proved inefficient at restricting breast cancer growth in vivo when investigated with xenograft and PDX models in immunodeficient mice [18, 19]. MCL-1 is known to play essential roles in a number of haematopoietic cell types [37]. Moreover, MCL-1-specific BH3-mimetic drugs can have higher affinity for human versus mouse MCL-1 meaning that xenograft studies may show efficient BH3-mimetic engagement of MCL-1 in the human-derived tumour at a given dose, whilst insufficiently targeting MCL-1 within the tumour microenvironment. Anti-tumour effects of MCL-1 inhibition could also occur through pro-tumour immune cell depletion and cancer associated fibroblasts and such contributions to tumour suppression may only emerge in homologous experimental systems [38, 39]. Here, using genetic and pharmaceutical methods on mouse tumours in immune proficient mice, we find that targeting MCL-1 can have single-agent inhibitory effect on mammary tumour growth of established mammary tumours. Preclinical studies demonstrating the therapeutic effect of targeting MCL-1 in haematopoietic tumours have supported the clinical application of MCL-1-specific BH3-mimetics: genetic deletion of *Mcl1* potently restricts tumour expansion and enhances survival in mouse models of AML, B-cell lymphoma and T-cell lymphoma [40–42]. The utility of targeting MCL-1 alone in solid tumours was recently demonstrated in lung adenocarcinoma where deletion of *Mcl1* restricted tumour development and treatment with S63845 shown to delay tumour progression in vivo [43]. Together with the present study, this validates the further investigation of drugs targeting MCL-1 in solid tumours.

In vivo dosing of S63845 at 25 mg/kg, unlike complete *Mcl1* deletion, is well tolerated by normal tissues and our study suggests a therapeutic window for efficacy of MCL-1 inhibitors in the clinic. In line with this, acute induction of whole body *Mcl1* haploid loss restrained the growth of established mammary tumours and extended survival. Furthermore, homozygous loss of *Mcl1*, specifically in the tumour epithelium, provoked tumour regression and allowed long-term tumour-free survival. It is worth noting that the context in which we find single-agent effect of S63845 in vivo has both an intact immune system and utilised higher dosing of S63845 (twice weekly 25 mg/kg v once weekly 25 mg/kg) when compared to related xenograft studies that saw no effect of S63845 alone but pronounced effects upon combination with docetaxel, trastuzumab or olaparib [18, 19]. We did not see this combination effect in an *MMTV-PyMT* allograft mouse model with once weekly S63845 and docetaxel. Given the lower potency of S63845 for mouse versus human MCL-1 it may be possible that the therapeutic index is underestimated in this context [17]. Therefore, the tumour suppressive effects of S63845 observed here could be due to increased frequency of dosing and/or a combination of tumour cell and microenvironment effects, and future studies will address the contribution of these factors [39]. It is also possible that whilst non-canonical functions of MCL-1 are not critical in the *MMTV-PyMT* model, these roles are required in the xenograft models tested previously.

In contrast to its critical role in *MMTV-PyMT* tumour growth in vivo, we were intrigued by the dispensability of *Mcl1* for tumour cell line growth in 2D monolayers in vitro where cell viability was jointly maintained by MCL-1 and BCL-XL. As a role for MCL-1 has been suggested in breast cancer stem cells we investigated growth in tumourspheres, known to measure breast cancer stem cell activity. Despite indifference to loss or inhibition of MCL-1 in 2D culture, the impact of targeting MCL-1 in a tumoursphere assay was dramatic that suggests that the biological relevance of targeting MCL-1 in vivo may be underestimated by interpretation of human breast cancer cell line 2D culture [9, 12, 34].

The impact of acute homozygous *Mcl1* deletion in fully developed mammary tumours was notable with regression occurring in almost all mice and long-term tumour-free survival achieved in 4/9 cases. Intriguingly, we found that tumour regression was more penetrant with genetic deletion of MCL-1 rather than pharmaceutical inhibition. This could indicate a non-apoptotic function of MCL-1 that is not inhibited using BH3-mimetic drugs such as increased mitochondrial respiration by an inner-membrane localised form of MCL-1 [13, 21]. We therefore investigated the ability of MCL-1 deletion to suppress *MMTV-PyMT* tumour growth when the downstream apoptotic effectors BAX/BAK were reduced. In this context there was no effect of MCL-1 loss on tumour growth indicating that the requirement for MCL-1 in sustained tumour growth is due to its known anti-apoptotic function within the BCL-2 family. This is supported by tumoursphere assays, indicative of breast cancer stemness, where using either human or mouse tumour cells we found that MCL-1 inhibition with BH3-mimetic drugs completely recapitulated the effect of MCL-1 deletion. Furthermore, reduction in BAX/BAK ablated the negative impact of both genetic and pharmaceutical targeting of MCL-1 in breast cancer stem cells. Together these data reveal that the pro-tumour role of MCL-1 in established breast cancer is predominantly due to its function in apoptosis regulation within the BCL-2 family. Whilst other non-apoptotic roles of MCL-1 could still be important in breast cancer, its function within the BCL-2 family is crucial and can be targeted by MCL-1-specific BH3-mimetic drugs in development for clinical use. Breast cancer stem cells are known to be responsible for recurrence and resistance to therapy; therefore, the requirement for MCL-1 in breast cancer stem cell activity, the regression of established tumours upon loss of MCL-1 and the association between high *MCL1* and stemness markers across a range of breast cancer patients indicate that targeting MCL-1 could be harnessed to improve breast cancer outcome.

## Materials and methods

### Mice and in vivo treatments

Animals were housed in a barriered facility proactive in environmental enrichment. All work was carried out in line with the Animals (Scientific Procedures) Act 1986 and the EU Directive 2010 and was sanctioned by the local ethical review process (University of Glasgow). *RosaCRE-ER*^*T2*^, *MMTV-PyMT, Mcl1tm3Sjk* (*Mcl1*^*fl/fl*^) (The Jackson Laboratory, ME, USA), and *ROSA-tdRFP* mice (acquired from the European Mouse Mutant Archive) and have all been described previously [44–47]. All mice had been backcrossed >10 generations FVB/N and all controls were littermates. Mice were monitored two to three times per week for tumour development. Tumour growth was monitored by calliper measurement three times per week, by staff blinded to outcome, and volume calculated using the equation ([length × width^2^]/2). Clinical endpoint was 15 mm diameter, at endpoint, mice were euthanized and tumours and lungs weighed. For orthologous tumour fragment transplant experiments single tumours were harvested from GEMM, washed in PBS and chopped by scalpel into 2 mm fragments immediately prior to transplantation into the fourth mammary fat pad of 7- to 10-week-old female FVB/N recipients (Charles River, UK). For orthologous transplantation of cell lines, 0.5 million cells in 50 µl 1:1 PBS:matrigel mix were transplanted into the fourth mammary fat pad of 7- to 10-week-old female FVB/N recipients (Charles River, UK). In vivo induction of CRE-ER^T2^ was achieved with tamoxifen (Sigma) 10 mg/ml stock in 200 µl sunflower seed oil by IP injection for 4 consecutive days. For in vivo dosing, S63845 (Active Biochem) was prepared as described [48] in 2% vitamin E/d-α-tocopheryl polyethylene glycol 1000 succinate (Sigma) immediately prior to IV administration by tail vein injection at 25 mg/kg and docetaxel (Merck) was dissolved in PBS and administered by intraperitoneal injection at 7.5 mg/kg. Mice were allocated to treatment arms when tumour diameter reached ~5 mm and distributed into treatment arms to match mean tumour volume in a non-random manner. Sample sizes were based on our previous experience with these models.

### Cell lines and in vitro assays

MDA-MB-231 cells used in this study are the MDA-MB-231-luc-D3H2LN line (Covance), cell lines were authenticated and confirmed negative for mycoplasma by the Molecular and Advanced Technologies service at the Cancer Research UK Beatson Institute. CRISPR/Cas9 gene editing was achieved using LentiCRISPRv2 backbone (Addgene #52961) with guide sequences: murine BAX: 5′-CAACTTCAACTGGGGCCGCG-3′ and BAK: 5′-GCGCTACGACACAGAGTTCC-3′; human BAX: 5′-AGTAGAAAAGGGCGACAACC-3′, BAK: 5′-GCCATGCTGGTAGACGTGTA-3′ and MCL1: 5’-GTATCACAGACGTTCTCGTA-3’. Lentiviral production, cell infection and selection were performed as previously described [49]. Cell lines were not cloned and pooled populations used in all experiments. For dual targeting of *Bax/Bak* in murine cells viral supernatant from LentiCRISPRv2_puro_ Bak was applied to target cells at 46 and 70 h post transfection being sequentially replaced with viral supernatant from LentiCRISPRv2_blasti_ Bax at 56 and 80 h post transfection. Co-selection with 1 µg/ml puromycin and 10 µg/ml blasticidine commenced at 94 h with selection media being replaced every 2–3 days for 1 week until all cells in uninfected control plates (puromycin, blasticidine and puromycin + blasticidine conditions) had died. For triple targeting of *MCL1/BAX/BAK* in human cells, blasticidine encoding vectors for *BAX/BAK* were used as described above to first generate BAX/BAK knockout line. The procedure was then repeated with LentiCRISPRv2_puro_ MCL1 to derive MCL1/ BAX/BAK triple targeted lines. Empty vector LentiCRISPRv2_blasti_ and LentiCRISPRv2_puro were used for control lines. All experiments were performed using <20 passage cells. For *MMTV-PyMT* cell line derivation, tumours were excised, washed in PBS, then finely chopped by scalpel prior to washing and seeding in media comprised of DMEM, 10% FBS, 2 µM L-glutamine, 50 U/ml penicillin, 50 µg/ml streptomycin (all Life Technologies), 5 µg/ml insulin (Life Technologies), 10 ng/ml EGF (Sigma) and 10 ng/ml cholera toxin. Primary tumour cells were considered cell lines following five successive passages as monolayers. In vitro induction of CRE-ER^T2^ was achieved with 100 nM 4-hydroxytamoxifen treatment prior to cell seeding for each experiment. YEJ2.1g-iRFP cells are an FVB *MMTV-PyMT* cell line that has been transduced with pBABE_iRFP_IRES_puro [50].

For tumoursphere assay single-cell suspension was prepared and 1000 cells plated per well in ultra-low attachment 24 well plates (Corning) in DMEM, 2 µM L-glutamine, 50 U/ml penicillin, 50 µg/ml streptomycin, 1X B27 (all Gibco, UK), 20 ng/ml EGF, 20 ng/ml FGF2 and 4 µg/ml Heparin (all Sigma) and tumourspheres >100 μm counted 7 days later. For viability assays 15,000 cells/well were seeded in 24 well plates and the following day the indicated treatments were added along with SYTOX Green (Invitrogen, UK) and imaged by Incucyte FLR (Essen Bioscience, UK). CD at 24 h post treatment was calculated using the equation CDtreatment/CDbasal where CDtreatment is SYTOX Green cells/cell confluence following 24 h treatment and CDbasal is SYTOX Green cells/cell confluence in control samples at 0 h. BH3-mimetics for in vitro experiments were ABT737, ABT199, WEHI539, A1210477 and S63845 (all ApexBio, UK). In vitro cell lysates for western blot were prepared in RIPA buffer with HALT Protease Inhibitor Cocktail (both Thermo Scientific, UK) and tumour lysates prepared from excised tumour fragments in the same buffer using a Precellys homogeniser according to manufacturer’s instructions (Bertin, France). Proteins were resolved by western blot on NuPage Bis-Tris 4–12% gradient gels (Invitrogen, UK) and transferred to nitrocellulose membranes (Amersham, UK). Blots were probed with antibodies to MCL-1 (94296), BCL-XL (2762), BAX (2772), BAK (12105) α-TUBULIN (2125) (all Cell Signaling, UK) and β-ACTIN (A4700) (Sigma, UK). Secondary antibodies were IRDye 800CW Donkey Anti-Rabbit IgG or IRDye 680RD Donkey Anti-Mouse IgG (Li-cor) and visualised using a Li-cor Odyssey CLx. For immunohistochemistry antibodies to BAX (14796) and MCL-1 (94296) (Cell Signaling, UK) were used with Vectastain ABC (Vector Laboratories) used in conjunction with MCL-1 staining.

### Gene expression analysis

Levels of *MCL1* and a range of marker genes were assessed in publicly available gene expression datasets. Data for METABRIC [51] and TCGA [52] were downloaded from c-Bioportal, data from the SCAN-B [53] study were extracted from NCBI GEO using accession GSE96058 and the Cb17 dataset was generated as a compendium of 2999 breast tumours from 17 Affymetrix studies integrated with batch correction as described previously [54].

### Statistical analysis

Sample size (*n*) is indicated in the figure legends. Statistical significance between two experimental groups was calculated by unpaired *t* test, two-tailed and multiple experimental groups by one-way ANOVA with Tukey’s correction for multiple comparisons using GraphPad Prism version 6.0c (GraphPad Software, CA, USA). Kaplan–Meier survival curves of tumour-related survival were also plotted using GraphPad Prism version 6.0c and Mantel–Cox (Log-rank). Centre values on graphs are mean or median values and error bars are standard deviation or standard error of the mean as detailed for each graph in the figure legend.

## Supplementary information

Supplemental Figure Legends

Supplemental Figure 1

Supplemental Figure 2

Supplemental Figure 3

Supplemental Figure 4

Supplemental Figure 5
