# Supplementary material for: Breast cancer dependence on MCL-1 is due to its canonical anti-apoptotic function
Source: Cell Death Differ. 2021 Mar 31;28(9):2589–600. doi: 10.1038/s41418-021-00773-4 (PMC8408186; doi:10.1038/s41418-021-00773-4)
Supplement: Supplementary file 1 — Supplemental Figure Legends [file 41418_2021_773_MOESM1_ESM.docx]

**Supplementary Figure legends**

**Supplementary Figure 1. Relevant to figure 1.**

**A.** Time for tumour progression of *MMTV-PyMT* tumours (from 5mm) to endpoint (15mm), WT n=9, median 18 days or *Mcl1* HET n=7 median 23 days, p=0.0283 (unpaired t test). Graph shows mean ±SD where each data point represents an individual mouse. Mice are those depicted in the survival curve in Figure 1B.

**B.** Orthotopically transplanted *RosaCreER^T2^;MMTV-PyMT* tumour fragments carrying *WT* or *Mcl1^fl/fl^* alleles were transplanted into female FVB recipients and allowed to grow until ~ 5mm diameter before vehicle treatment, n=13 WT v n=7 *Mcl1^fl/fl.^* Error bars represent mean ±SD of percentage change in tumour volume 3 weeks post treatment n.s. = not significant, unpaired t test.

**C**. Kaplan Meier survival curve of mice carrying tumours shown in (B). Median survival 33 v 37 days P=0.3228 Log-rank (Mantel-Cox) test. Experiment was performed in parallel to that shown in main figures 1C-D and recipients were randomly assigned to vehicle or tamoxifen treatment groups when tumours reached ~ 5mm diameter. Donor tumours are as described in Figure 1C-D.

**Supplementary Figure 2. Relevant to figure 2.**

**A.** Diagram depicting derivation of *MMTV-PyMT;RosaCRE-ER^T2^;Mcl1^fl/fl^* cell lines utilised in Figures 2 and 3. Parental cell line was derived *in vitro* from a *MMTV-PyMT;RosaCRE-ER^T2^;Mcl1^fl/fl^* MCL-1 proficient (Tamoxifen naive) mammary tumour then transduced with CRISPR/Cas9 control vectors or CRISPR/Cas9 vectors targeting *Bax* and *Bak* and stable cell lines generated prior to orthologous transplantation assays shown in figures 2 and 3.

**B.** Graph showing mean change in tumour volume of individual tumours from Figures 2B-C. Vehicle control n=7 mice (black lines) and S63845 n=10 mice (red lines), tumours were measured thrice weekly by calliper measurement and volume is expressed as weekly mean relative to volume at start of treatment.

**C-D.** *MMTV-PyMT* cell line allograft tumour growth and survival following reduced frequency of S63845 dosing and in combination with docetaxel. 0.5 million cells of YEJ2.1g-iRFP *MMTV-PyMT* cell line were transplanted into the mammary fat pad of female FVB mice and tumours allowed to grow to 5mm in diameter (designated day 0) before assignment to treatment arm, n=5 mice per condition. Treatment schedule was docetaxel 7.5mg/kg or vehicle control IP on days 0,7,14,21 and S63845 25mg/kg IV or vehicle control on days 3,10,17 (**C**) Graph showing mean tumour volume of individual tumours at 2 weeks following treatment with docetaxel (blue) S63845 (red), both docetaxel and S63845 (purple) or vehicle control (black). Points represent mean volume (from thrice weekly calliper measurement) of individual tumours, bars represent mean of 5 mice per condition (value indicated on graph) ±SEM, no significant differences as determined by one-way ANOVA with Tukey’s correction for multiple comparisons.

**D.** Graph of survival from start of treatment of mice carrying tumours shown in (C). Points represent individual mice, bars represent mean of 5 mice per condition ±SD (values indicated on graph), no significant differences as determined by one-way ANOVA with Tukey’s correction for multiple comparisons,

**Supplementary Figure 3. Relevant to figure 3.**

**A.** Western blot of *MMTV-PyMT;RosaCRE-ER^T2^;Mcl1^fl/fl^* cell line with CRISPR/Cas9 targeting of *Bax* and *Bak*, actin panel loading control is shown.

**B.** Western blot showing MCL-1 expression in endpoint tumours from vehicle (CRE inactive), n=5 or tamoxifen (CRE activated), n=4 tumours arising from transplantation of *MMTV-PyMT;RosaCRE-ER^T2^;Mcl1^fl/fl^;CRISPR;Bax/Bak* cell line. Tumour material sampled for western blot will include host-derived (WT for *Mcl1*) stroma and donor-derived tumour epithelium. 9 tumours were evaluated along with molecular weight marker on a 10 well gel. Relative MCL-1 expression is calculated relative to ACTIN for each tumour and then expressed relative to the tumour loaded furthest to the left. These values are used in main figure 3D.

**C.** IHC of BAX in endpoint tumours from (i) *MMTV-PyMT;RosaCRE-ER^T2^;Mcl1^fl/fl^;CRISPR;Control* cell line (ii) *MMTV-PyMT;RosaCRE-ER^T2^;Mcl1^fl/fl^;CRISPR;Bax/Bak* cell line. Representative of n=4 tumours, scale bar indicates 200 μm.

**D.** IHC of MCL-1 in endpoint tumours from (i) Vehicle treated (CRE inactive) and (ii) Tamoxifen treated (CRE activated) *MMTV-PyMT;RosaCRE-ER^T2^;Mcl1^fl/fl^;CRISPR;Bax/Bak* cell line. Representative of n=2 tumours, scale bar indicates 200 μm.

**Supplementary Figure 4. Relevant to figure 4.**

**A.** Additional independent *MMTV-PyMT;RosaCRE-ER^T2^;Mcl1^fl/fl^* tumour cell line (8.2a) to that shown in Figure 4B. Induction of *Mcl1* deletion with 100 nM 4-OHT at time of plating, medium changed 24h later and 1 μM of indicted BH3 mimetic drugs added. Viability at 24h following BH3 mimetic treatment is expressed as Sytox/Confluence relative to control. Graph shows mean ±SD, no significant differences as determined by one-way ANOVA with Tukey’s correction for multiple comparisons, n=3 independent experiments).

**Supplementary Figure 5. Relevant to figure 5.**

**A.** Tumoursphere assay with MDA-MB-231 CRISPR/Cas9 control cell line, DMSO vehicle (Control), S63845 (1 μM or 5 μM) or A1210477 (1 μM or 5 μM) were added at time of plating and spheres were counted at day 7. Points represent independent experiments expressed as % tumoursphere formation relative to control. Bars represent mean ±SD of 3 (1 μM conditions), 4 (5 μM conditions) or 14 (Control) biological replicates. One-way ANOVA analysis with Tukey’s correction for multiple comparisons, *P<0.05, **P<0.01, ****P<0.0001, n.s.= not significant.

**B.** Western blot of MDA-MB-231 cells with CRISPR/Cas9 targeting of *MCL1, BAX* and *BAK*, tubulin panel loading control is shown.

**C**. Correlation of *MCL1* expression with *VIM, SNAI2, CDH3, CD44* and *ALDH1A1* in METABRIC breast cancers (1904 tumours, METABRIC (52)) PAM50 subtype and ER negative status indicated in top rows.

**D**. Comparison of *VIM, SNAI2* and *ALDH1A1* expression in low *MCL1* (lower quartile) and high *MCL1* (upper quartile) with ER status indicated (red dots for ER negative, blue dots for ER positive tumours) METABRIC dataset as in B.

**E.** Correlation of *MCL1* expression with *VIM, SNAI2, CDH3, CD44* and *ALDH1A1* in Lund breast cancers (3273 tumours, extracted from NCBI GEO using accession GSE96058 (53) *Pearson correlation P<0.05.

**F.** Correlation of *MCL1* expression with *VIM, SNAI2, CDH3, CD44* and *ALDH1A1* in Cb17 breast cancers (2999 tumours, (54).

*Pearson correlation P<0.05.
