# Supplementary figures and images for: Breast cancer dependence on MCL-1 is due to its canonical anti-apoptotic function
Source: Cell Death Differ. 2021 Mar 31;28(9):2589–600. doi: 10.1038/s41418-021-00773-4 (PMC8408186; doi:10.1038/s41418-021-00773-4)

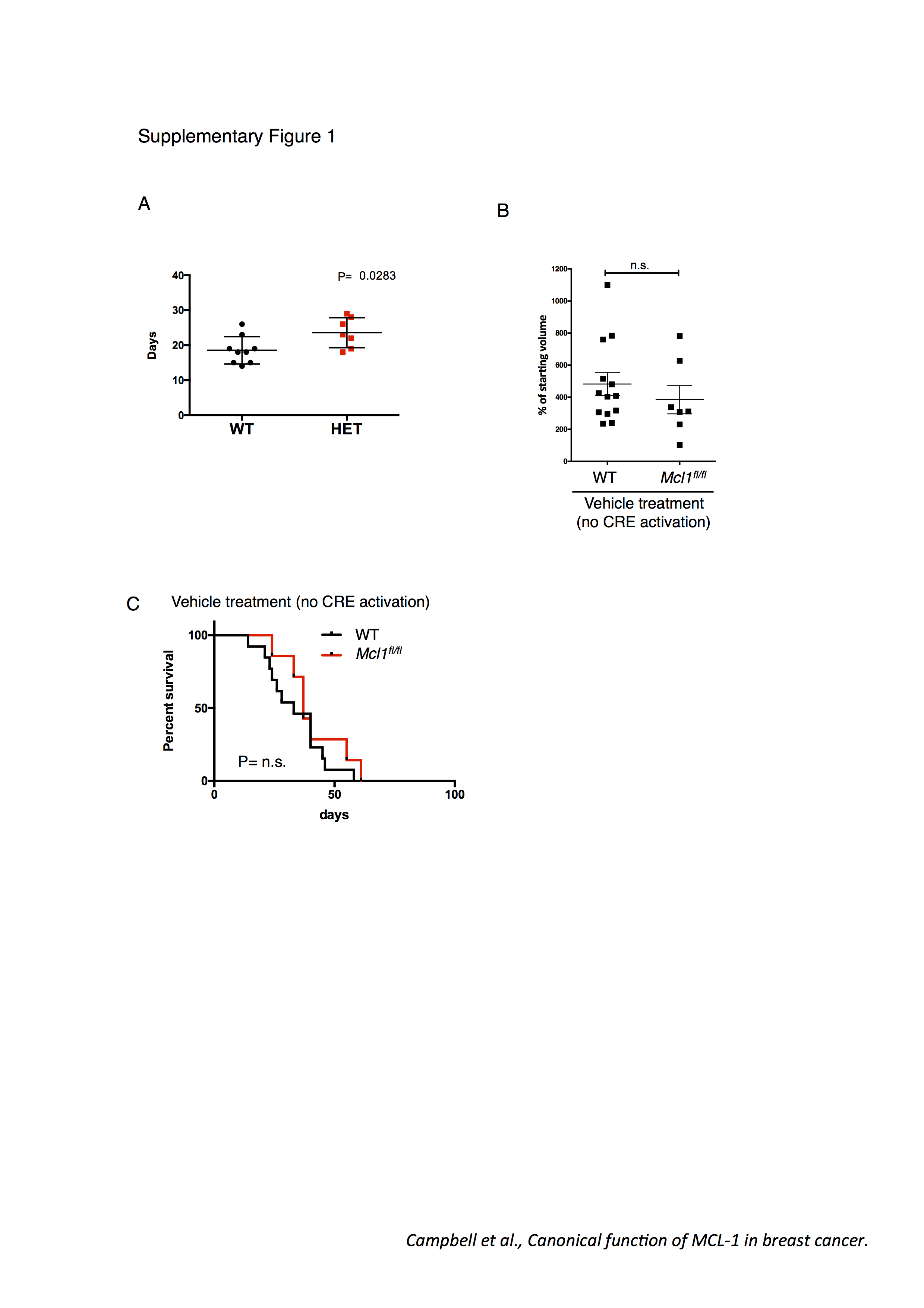

Supplement: Supplementary file 2 — Supplemental Figure 1 [file 41418_2021_773_MOESM2_ESM.tif]

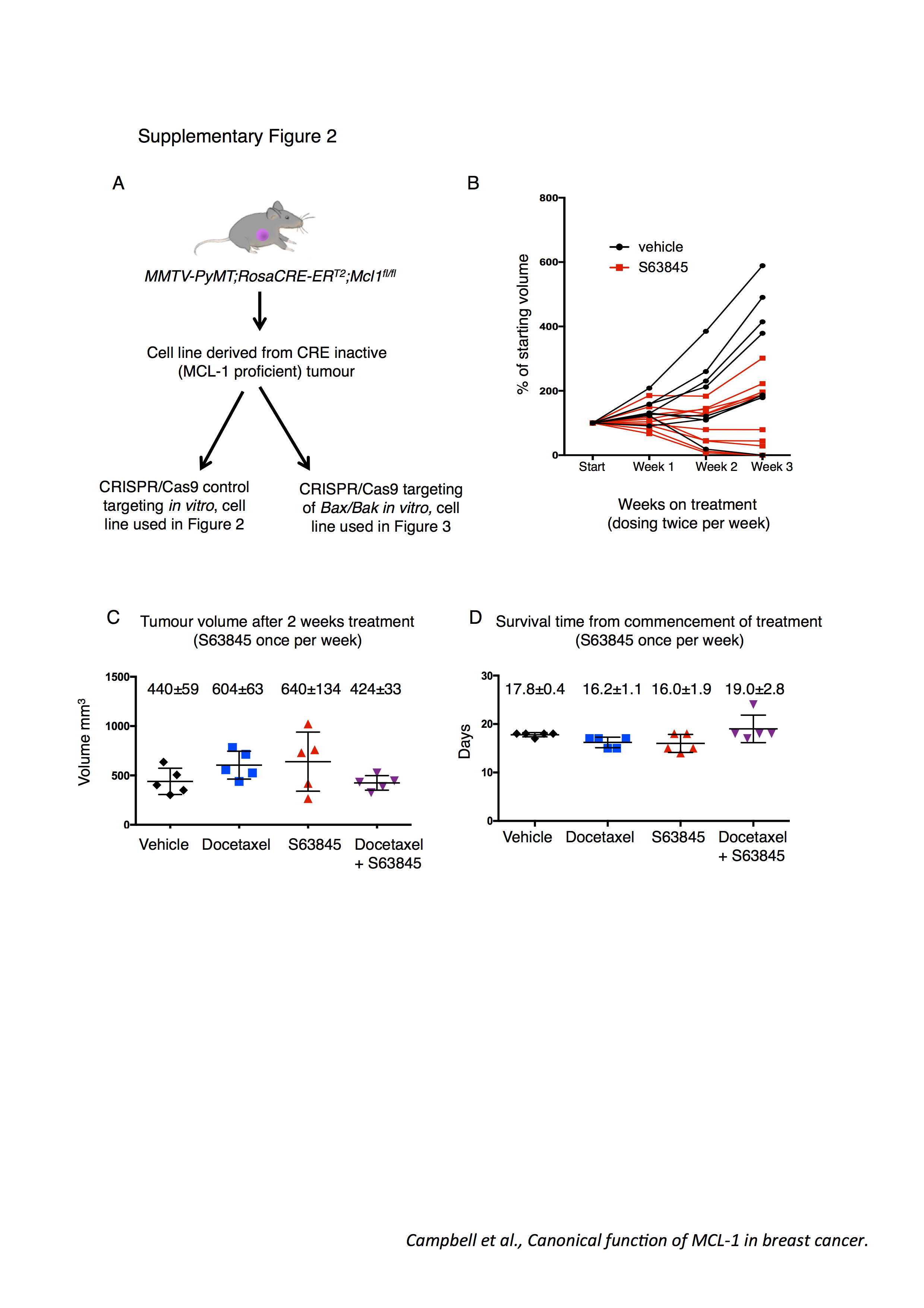

Supplement: Supplementary file 3 — Supplemental Figure 2 [file 41418_2021_773_MOESM3_ESM.tif]

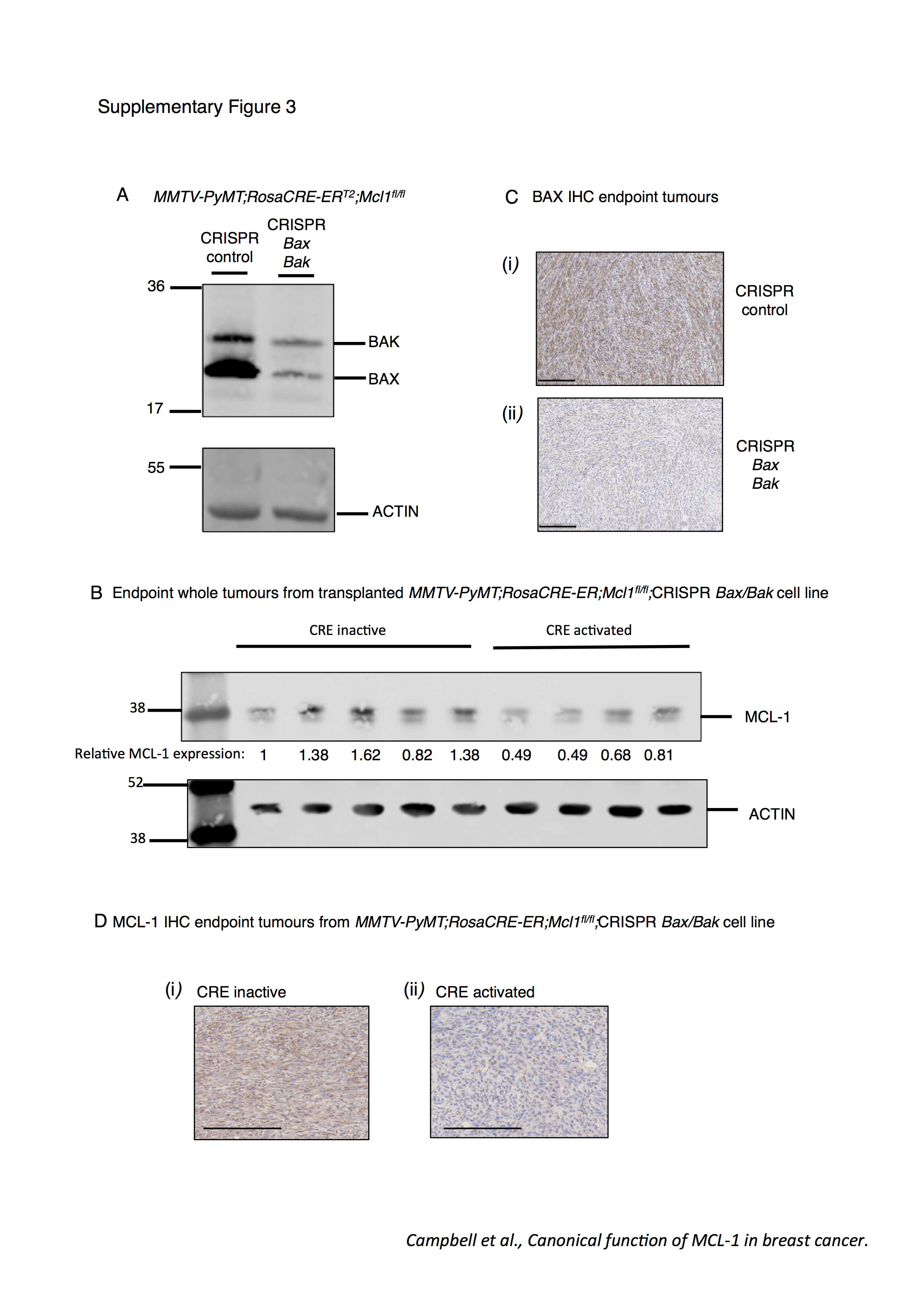

Supplement: Supplementary file 4 — Supplemental Figure 3 [file 41418_2021_773_MOESM4_ESM.tif]

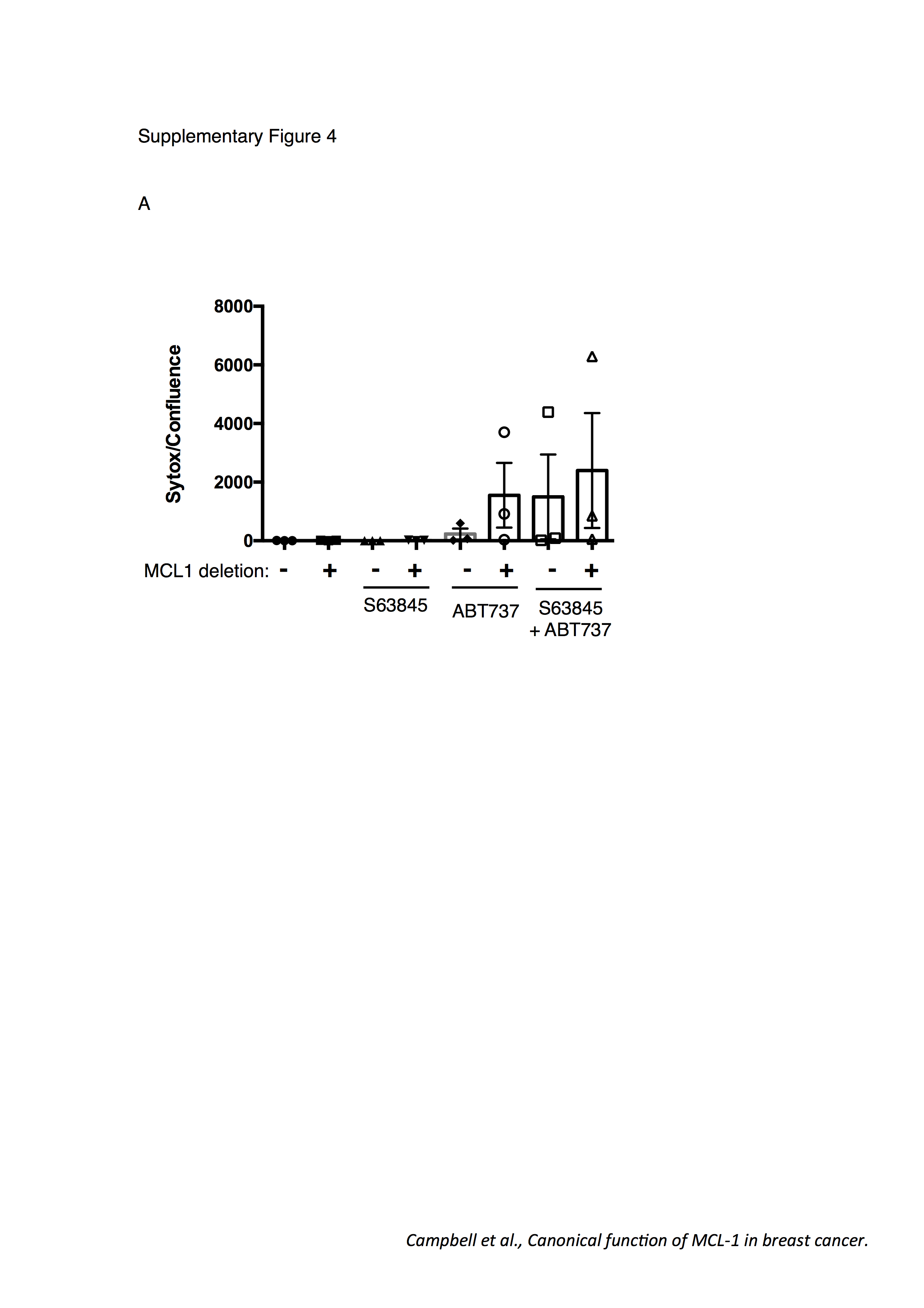

Supplement: Supplementary file 5 — Supplemental Figure 4 [file 41418_2021_773_MOESM5_ESM.tif]
